# The Importance of the Mining Subsidence Reservoirs Located Along the Trans-Regional Highway in the Conservation of the Biodiversity of Freshwater Molluscs in Industrial Areas (Upper Silesia, Poland)

**DOI:** 10.1007/s11270-015-2445-z

**Published:** 2015-05-19

**Authors:** Iga Lewin, Aneta Spyra, Mariola Krodkiewska, Małgorzata Strzelec

**Affiliations:** Department of Hydrobiology, Faculty of Biology and Environmental Protection, The University of Silesia, 9 Bankowa Street, 40-007 Katowice, Poland

**Keywords:** Mollusc community, Rare species, Transportation routes, Anthropogenic reservoirs, *Anodonta cygnea*, Environmental factors

## Abstract

The objectives of the survey were to analyse the structure of the mollusc communities in the mining subsidence reservoirs that were created as a result of land subsidence over exploited hard coal seams and to determine the most predictive environmental factors that influence the distribution of mollusc species. The reservoirs are located in urbanised and industrialised areas along the Trans-Regional Highway, which has a high volume of vehicular traffic. They all have the same sources of supply but differ in the physical and chemical parameters of the water. In total, 15 mollusc species were recorded including four bivalve species. Among them *Anodonta cygnea* is classified as Endangered according to the Polish Red Data Book of Animals and also as Near Threatened according to the European Red List of Non-marine Molluscs. Eleven of the 15 mollusc species are included on the European Red List of Non-marine Molluscs as Least Concern. Conductivity, pH and the concentration of calcium were the parameters most associated with the distribution of mollusc species. Canonical correspondence analysis showed that *Potamopyrgus antipodarum*, *Radix balthica*, *Physella acuta*, *Gyraulus crista* and *Pisidium casertanum* were associated with higher conductivity and lower pH values. *A. cygnea*, *Anodonta anatina* and *Ferrissia fragilis* were negatively influenced by these parameters of the water. The results of this survey showed that the mining subsidence reservoirs located in urbanised and industrialised areas provide refuges for rare and legally protected species and that they play an essential role in the dispersal of alien species as well.

## Introduction

The development of transport, including the construction of roads, strongly interferes with the natural environment and causes dangerous changes in aquatic ecosystems. These are mostly dependent on the volume of traffic and are caused not only by the noise and pollution that come from vehicles but also by the impact of the different elements of road infrastructure on the environment (Sriyaraj and Shutes 2001; Kołodziejczyk et al. 2009). During the construction of roads, the surface of the water reservoirs that are located along transportation routes is reduced. Reservoirs that are located near to the roads that are being constructed are rebuilt and converted into storage or infiltration reservoirs, and they remain under the influence of traffic.

The impacts of roads on water bodies are diverse and the most important, apart from the traffic, include, e.g. the presence of denivelation in the form of road embankments and excavations and open drainage ditches (Barrett et al. 1998; Lu et al. 2009) which constitute serious impediments to the occurrence of aquatic vegetation. The source of the impacts that are associated with the formation of impurities include roadsides, slopes, engineering facilities and other objects that are associated with roads (e.g. gas stations, services areas and parking). Emissions along transport routes with heavy traffic as well as dry deposits and atmospheric precipitation that are rich in dust of automotive and industrial origin also play important roles (Sriyaraj and Shutes 2001). The reasons for the negative impact of road infrastructure on surface water and groundwater are various pollutants such as organic compounds, heavy metals, fumes, dust from the abrasion of tires and substances that are used for de-icing road surface (Wang et al. 2003; Pawluk et al. 2011), which pass into the water reservoirs with surface runoff despite the use of different types of infiltration devices and devices for the retention of rainwater runoff. The accumulation of heavy metals in the bottom sediments of anthropogenic water bodies, lakes and rivers can lead to the hazardous and highly unpredictable contamination of natural ecosystems (Dmochowski and Dmochowska 2011), which affects the occurrence of freshwater benthos fauna, especially gastropods and bivalves. The construction of the roads in wetland areas also causes the massive destruction of the habitats of amphibians due to the movement and storage of redundant soils (Kurek et al. 2011). These impacts are compounded when the distance between the water bodies and the road is small (less than 200 m) or when they are located along the road like the mining subsidence reservoirs.

Anthropogenic water bodies, especially subsidence ponds, originate on urban areas that have been impacted by mineral extraction, industrialisation, urbanisation and the development of additional land uses (Drecker et al. 1995; Álvarez-Fernández et al. 2005; Wang et al. 2008; Zealand and Jeffries 2009). Land subsidence continues on sites that are still impacted by heavy industry (e.g. southern Poland and southeast Northumberland), which causes natural aquatic environments to disappear when new water reservoirs are created (Meng et al. 2009; Jeffries 2012). Despite the fact that these water bodies are exposed to pollution, they create refuges for many species of plants and animals, which often include rare species (Wood et al. 2001; Wood et al. 2003). This is particularly important in the case of reservoirs that are located along the numerous, large transportation routes on which the high intensity of vehicular traffic causes these reservoirs to be vulnerable to contamination. Reservoirs along transportation routes may provide the only breeding habitats for amphibians, dragonflies and molluscs as well as being refuges in human-dominated landscapes for rare species and those that are protected by law (Le Viol et al. 2012; Jeanmougin et al. 2014). Many water bodies that were developed for technical anthropogenic uses are also refuges for biodiversity, but their potential roles have not yet been seriously investigated.

The objectives of our survey were to analyse the structure of the mollusc communities in the mining subsidence reservoirs located along the transportation routes in an industrial area in terms of the number of species, density, dominance, constancy pattern and biodiversity; to determine which environmental factors influence the structure of mollusc communities; and to evaluate the ecological conservation value of the mining subsidence reservoirs that support molluscs including rare, threatened and alien species.

## Materials and Methods

### Study Area

Upper Silesia is the most urbanised and industrialised region in Poland. This area constitutes one of the largest coal basins in the world. Upper Silesia has almost no natural water bodies; anthropogenic reservoirs, including mining subsidence reservoirs, are the most common.

**Fig. 1:**
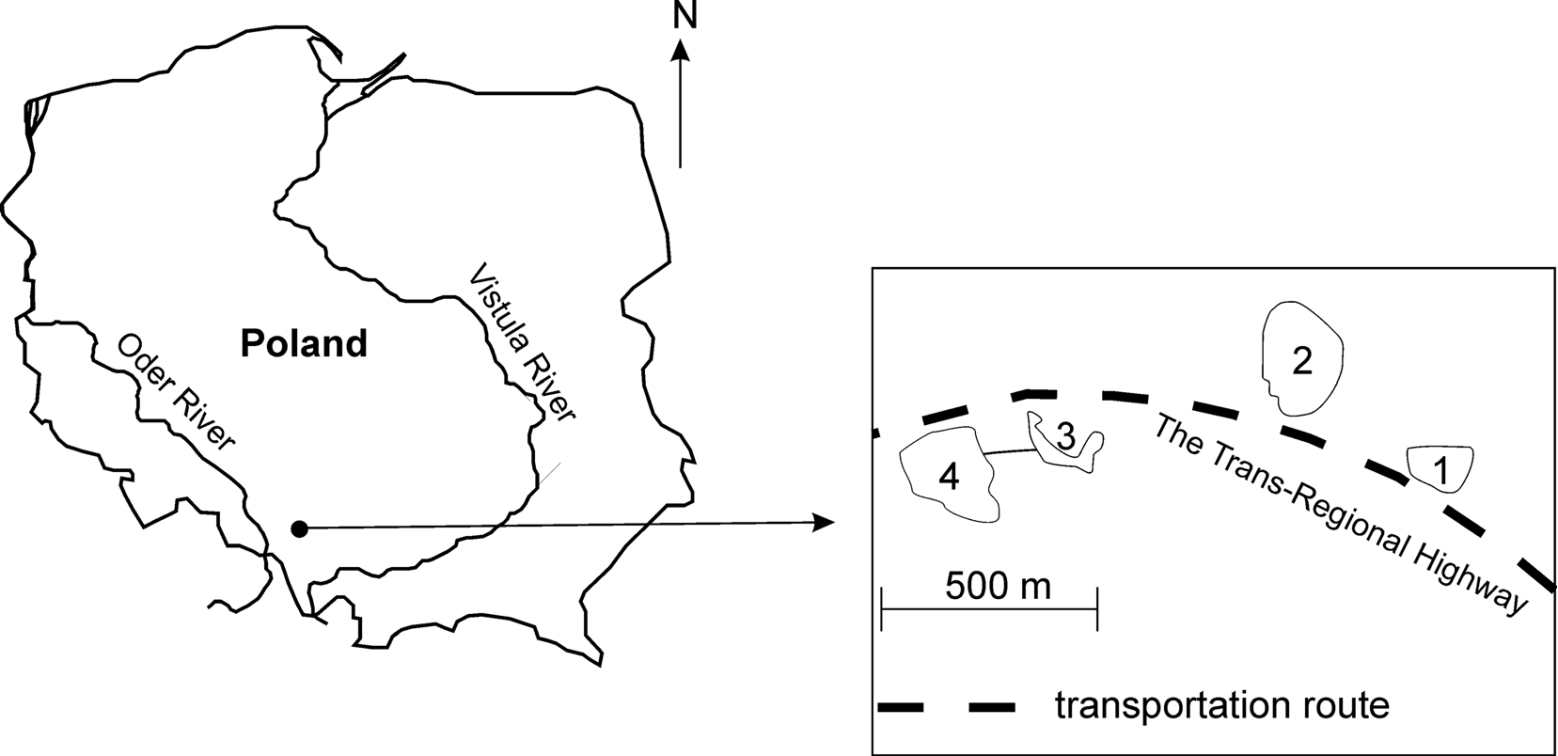
Location of the mining subsidence reservoirs along the Trans-Regional Highway (*T*-*R H*)

The study was carried out from 2009 to 2011 (three times each year in May, August and October) in four mining subsidence reservoirs (Upper Silesia, Southern Poland) that were created as a result of the land subsidence over exploited hard coal seams (Fig. 1). These reservoirs are located along the Trans-Regional Highway (T-R H), which connects the cities in the agglomeration that have a high volume of vehicular traffic in urbanised and industrialised areas. The reservoirs all have the same sources of supply (Table 1).

**Table 1:** Characteristics of the mining subsidence reservoirs located along the Trans-Regional Highway (*T*-*R H*)

	Mining subsidence reservoirs			
	1	2	3	4
Geographic coordinates	50° 17.686′	50° 17.893′	50° 17.701′	50° 17.784′
	18° 55.505′	18° 55.391′	18° 54.597′	18° 54.720′
Elevation (m a.s.l.)	275	272	275	268
Surface area (ha)	1.7	4.5	2.5	4.5
Max. depth (m)	3.5	4.0	6.0	3.5
Source of water supply	Atmospheric precipitation, surface runoff, groundwater			
Reservoir management	Stocked with fish, wildfowl	Stocked with fish, wildfowl, recreation	Stocked with fish, wildfowl	Stocked with fish

### Sampling Procedure

The samples were collected at a distance of 1 m from the banks of the reservoirs. The depth of collecting samples ranged from 20 to 70 cm. The samples of molluscs were collected according to quantitative methods by placing a quadrat frame (25 × 25 cm) randomly on the substratum of the mining subsidence reservoirs. Sixteen subsamples (one frame each) were taken at each sampling site, which in total constituted one sample (1 m^2^) and included bottom sediments up to 5 cm, macrophytes and surface water. A D-frame net with a sharp cutting edge and mesh size of 465 μm was used for the sampling area that was enclosed within each quadrat frame. The entire sampling area (the area within which the quadrat frame was placed) measured to about 3 m^2^.

The collected material was transported to the laboratory in plastic containers. The samples were washed using a 0.5-mm mesh sieve and then preserved in 75 % ethanol. Molluscs were identified to the species level based on their morphological and anatomical features according to Glöer and Meier-Brook (1998), Glöer (2002) and Jackiewicz (2000). The nomenclature follows Glöer and Meier-Brook (1998) and Glöer (2002). The density of molluscs was estimated as the number of individuals per square metre. Samples of water and bottom sediments were collected from each mining subsidence reservoir before the sampling of molluscs. Analyses of physical and chemical parameters of the water—temperature, conductivity, total dissolved solids and pH—were measured in the field using a portable HI 9811–5 meter (Hanna Instruments) and dissolved oxygen using a CO-401 oxygen meter (Elmetron). Analyses of ammonium, nitrite, nitrate, phosphate, calcium and chlorides, sulphates and iron concentration in the water, hardness and alkalinity were carried out in the laboratory using standard methods (Hermanowicz et al. 1999). Organic matter content in the bottom sediments was determined by the loss on ignition (LOI) method, which measures weight loss in bottom sediment samples after burning at 550 °C according to PN-88/B-04481 (Myślińska 2001). Macrophytes were recorded at the sampling sites and identified to species according to Szafer et al. (1986).

### Calculation of Indices and Statistical Analysis

The structure of the mollusc communities was analysed according to following indices (Górny and Grüm 1981):

1. Dominance (*D*%):
  $$ D=\frac{n_a}{n}\cdot 100 $$
  where
  *n*_*a*_ – the number of individuals of species *a*,
  *n* – the total number of individuals in the sample.
  The value of the dominance index *D* was divided into five classes:
  eudominants *D* > 10.0 % of sample, dominants *D* = 5.1–10.0 % of sample, subdominants *D* = 2.1–5.0 % of sample, recedents *D* = 1.1–2.0 % of sample and subrecedents *D* ≤ 1.0 % of sample.
2. The Shannon-Wiener index (*H*’):
  $$ {H}^{\prime }=-{\displaystyle \sum_{i=1}^S{P}_i \ln {P}_i} $$
  where
  ${P}_i=\frac{N_i}{N}$ – the proportion of individuals of species *i*

Canonical ordination analyses to relate the species composition of the molluscs to environmental variables were carried out using CANOCO for Windows version 4.5 (Ter Braak and Šmilauer 2002). The following environmental variables were included into the analysis: the physical and chemical parameters of the water, the organic matter content (%) in the bottom sediments and the number of macrophyte species.

The appropriate type of analysis was chosen to analyse the species data using detrended correspondence analysis (DCA) and the length of the gradient. Preliminary DCA on the biological data revealed that the gradient length exceeded 4 SD (the standard deviation), thus indicating that the biological data exhibited a strong unimodal response to underlying environmental variables. Therefore, a unimodal direct ordination canonical correspondence analysis (CCA) with a forward selection was used for the reduction of the large set of environmental variables. The statistical significance of the relationship between the biological data and environmental variables was evaluated using the Monte Carlo permutation test (499 permutations). Both the biological and environmental data were log-transformed (Ter Braak and Šmilauer 2002). The CCA was performed based on pooled samples (3 seasons × 3 years × 4 mining subsidence reservoirs) and the density of mollusc species (biological data).

The significance of the differences in the values of the environmental variables as well as in the density of Mollusca and the number of species between the mining subsidence reservoirs was calculated using the Kruskal-Wallis ANOVA and a multiple comparison post hoc test (Statistica program version 9.0). The value of the environmental variables and biological data did not reveal a normal distribution, and this justified the use of a non-parametric test. The values of the Shannon-Wiener index *H*’ (the natural logarithm base) were calculated using the MVSP program version 3.13p (Kovach Computing Services).

## Results

### The Physical and Chemical Parameters of the Water and Organic Matter Content in the Bottom Sediments

Data summarising the physical and chemical parameters of the water and the organic matter content in the bottom sediments in the mining subsidence reservoirs are given in Table 2. The organic matter content in the bottom sediments ranged from 1.2 to 28.2 %. The temperature of the water ranged from 5.6 to 25.5 °C. The highest conductivity of up to 1920 μS cm^−1^ was recorded in mining subsidence reservoirs 3 and 4 (Table 2). The highest concentration of nutrients excluding the concentration of ammonium was recorded in mining subsidence reservoir 1. The hardness, alkalinity, concentration of calcium, chlorides and sulphates were relatively high or very high in reservoirs 3 and 4. The Kruskal-Wallis ANOVA test (*df* = 3) revealed statistically significant differences in the median concentration of dissolved oxygen (*H* = 8.39, *p* = 0.0390), pH (*H* = 16.68, *p* = 0.0008), conductivity (*H* = 21.62, *p* = 0.0001), total dissolved solids (*H* = 21.62, *p* = 0.0001), chlorides (*H* = 20.40, *p* = 0.0001), alkalinity (*H* = 15.66, *p* = 0.0013), hardness (*H* = 19.50, *p* = 0.0002) and calcium (*H* = 23.14, *p* < 0.0001), sulphates (*H* = 17.90, *p* = 0.0005) and iron (*H* = 15.31, *p* = 0.0016) at the sampling sites between the mining subsidence reservoirs.

**Table 2:** The physical and chemical parameters of the water (ranges) and organic matter content (%) in the bottom sediments in the mining subsidence reservoirs

Parameter	Mining subsidence reservoirs			
	1	2	3	4
Temperature (°C)	8.4–24.3	8.3–24.0	5.6–25.3	9.3–25.5
Conductivity (μS cm^−1^)	450–1050	330–410	1790–1920	1700–1920
Total dissolved solids (mg dm^−3^)	220–520	160–200	890–960	850–960
pH	7.5–8.2	8.5–8.8	7.2–8.1	7.8–8.4
Dissolved oxygen (mg O_2_ dm^−3^)	4.4–7.7	3.9–10.0	5.2–6.7	6.2–9.0
Ammonium (mg NH_4_^+^ dm^−3^)	0.01–0.29	0.02–0.36	0.15–0.72	0.02–0.36
Nitrites (mg NO_2_^−^ dm^−3^)	0.03–0.17	0.03–0.10	0.03–0.07	0.03–0.10
Nitrates (mg NO_3_^−^ dm^−3^)	3.01–20.77	2.66–8.42	1.77–14.62	0.44–7.97
Phosphates (mg PO_4_^3−^ dm^−3^)	0.02–2.56	0.03–0.36	0.01–1.17	0.08–0.33
Hardness (mg CaCO_3_ dm^−3^)	195–335	99–220	330–510	335–525
Calcium (mg Ca dm^−3^)	48–72	26–62	158–202	120–180
Alkalinity (mg CaCO_3_ dm^−3^)	150–215	95–140	175–270	150–270
Chlorides (mg Cl^−^ dm^−3^)	19–30	20–49	115–142	98–140
Sulphates (mg SO_4_^2−^ dm^−3^)	75–50	75–300	300–1140	300–1800
Iron (mg Fe dm^−3^)	0.10–0.55	0.05–0.08	0.11–0.13	0.10–0.23
Organic matter (%)	4.8–15.2	1.2–13.0	3.7–26.4	3.8–28.2

### Macrophytes

In total, 15 macrophyte taxa were recorded at the sampling sites in the mining subsidence reservoirs. The number of taxa ranged from 2 to 9 in individual reservoirs. *Najas marina*, which is typical in freshwater and brackish water, occurred at only one sampling site. *Nymphaea alba* is legally protected in Poland (Dz. U. 2014a). Only one taxon of algae was found in two of the four reservoirs (Table 3).

**Table 3:** The occurrence of macrophytes at the sampling sites in the mining subsidence reservoirs

Taxa	Mining subsidence reservoirs			
	1	2	3	4
*Calystegia sepium* (L.) R. Br.	X			
*Ceratophyllum demersum* L. S. Str.			X	
Cladophora sp.	X		X	
*Eleocharis palustris* (L.) Roem. & Schult.		X		
*Euptorium cannabinum* L.	X			X
*Glyceria maxima* (Hartm.) Holmb.	X			
*Lycopus europaeus* L.	X			
*Myriophyllum spicatum* L.		X		
*Najas marina* L.				X
*Nymphaea alba* L.	X			
*Phragmites australis* (Cav.) Trin. Ex. Steud.	X	X		X
*Potamogeton crispus* L.	X			X
Batrachium sp.				X
*Typha angustifolia* L.	X			
*Typha latifolia* L.				X
Total number of taxa	9	3	2	6

### Structure of Mollusc Communities

**Fig. 2:**
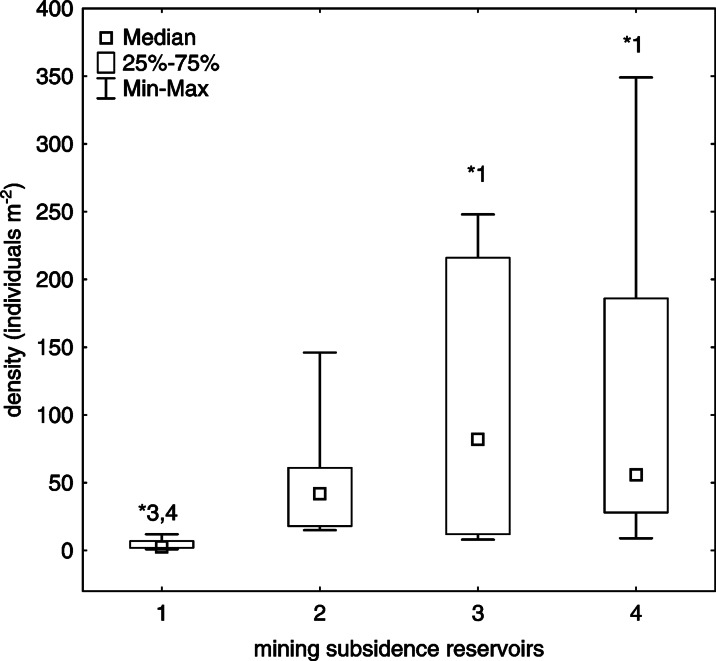
Box-and-whisker plot showing the density of molluscs in the mining subsidence reservoirs (medians, interquartile ranges, minimum and maximum values). *Asterisks* over a whisker denote significant differences between reservoirs (the Kruskal-Wallis ANOVA and multiple comparison post hoc test)

In total, 15 mollusc species were recorded in the mining subsidence reservoirs (Table 4). From 6 to 11 mollusc species were found in individual reservoirs. Only two species, *Radix auricularia* and *Gyraulus albus*, were recorded in all of the reservoirs. *R. auricularia* was eudominant (reservoirs 1, 2 and 4) or subdominant (reservoir 3), whereas *G. albus* was eudominant (reservoirs 1 and 3), dominant (reservoir 2) or subdominant (reservoir 4) in the mollusc community (Table 4). Four bivalve species occurred in the mollusc community. Among them, *Anodonta cygnea* is protected by Polish legislation (Dz. U. 2014b). Unionid species, i.e. *Unio pictorum*, *Anodonta cygnea* and *Anodonta anatina*, were subdominants or recedents in the mollusc community in reservoir 2. Unionid *Anodonta cygnea* was only recorded in the mollusc community in reservoir 2 (recedent). The fingernail clam, *Pisidium casertanum*, occurred in two reservoirs (eudominant or subdominant). Three gastropod species that are alien in Polish fauna, i.e. *Potamopyrgus antipodarum*, *Physella acuta* and *Ferrissia fragilis*, were also recorded (reservoirs 2, 3 and 4). *F. fragilis* was eudominant in the mollusc community in reservoir 2, whereas *Physella acuta* was eudominant in reservoir 4 (Table 4). The mean density of molluscs ranged from 10 to 123 individuals m^−2^. The maximum density of molluscs amounted to 349 individuals m^−2^ in reservoir 4. The median density was highest in reservoir 3 (Fig. 2). The Kruskal-Wallis ANOVA test revealed statistically significant differences in the median number of mollusc species (*H* = 11.82, *p* = 0.008) and density (*H* = 11.38, *p* = 0.0098) between the mining subsidence reservoirs. Multiple comparison post hoc tests showed statistically significant differences in the median number of species and density between certain reservoirs, i.e. reservoirs 1, 3 and 4 (Fig. 2). The mean values of the Shannon-Wiener index *H*’ ranged from 0.85 to 1.22. The species diversity as measured by the Shannon-Wiener index (*H*’) was highest in reservoir 3 (Table 4).

**Table 4:** The values of the dominance (*D*%) index calculated for the mollusc communities in the mining subsidence reservoirs located along the Trans-Regional Highway (*T*-*R H*)

Species	Mining subsidence reservoirs				Category of threat^a^
	1	2	3	4	
	*D*%	*D*%	*D*%	*D*%	
*Potamopyrgus antipodarum* (J. E. Gray, 1843)	–	–	0.8	2.4	
*Radix auricularia* (Linnaeus, 1758)	14.0	17.8	4.5	14.2	LC
*Radix balthica* (Linnaeus, 1758)	–	–	17.7	25.5	LC
*Lymnaea stagnalis* (Linnaeus, 1758)	–	–	5.8	–	LC
*Physella acuta* (Draparnaud, 1805)	–	–	–	42.6	
*Planorbarius corneus* (Linnaeus, 1758)	2.0	–	5.1	0.3	LC
*Ferrissia fragilis* (Tryon, 1863)	–	64.4	–	0.6	
*Planorbis planorbis* (Linnaeus, 1758)	8.0	–	28.6	–	LC
*Anisus spirorbis* (Linnaeus, 1758)	–	–	0.1	–	LC
*Gyraulus albus* (O. F. Müller, 1774)	18.0	7.9	25.8	4.0	LC
*Gyraulus crista* (Linnaeus, 1758)	8.0	–	11.6	4.9	LC
*Unio pictorum* (Linnaeus, 1758)	–	3.2	–	1.5	LC
*Anodonta cygnea* (Linnaeus, 1758)	–	1.6	–	–	NT
*Anodonta anatina* (Linnaeus, 1758)	–	4.8	–	0.2	LC
Anodonta sp.	–	0.3	–	–	
*Pisidium casertanum* (Poli, 1791)	50.0	–	–	3.7	LC
∑ of specimens	50	377	728	863	
No of taxa	6	7	8	11	
Mean density (individuals m^−2^)	10	54	121	123	
Shannon-Wiener index *H*’	0.91	0.85	1.22	1.14	

^a^According to the European Red List of Non-marine Molluscs (Cuttelod et al. 2011)

### Mollusc Communities in Relation to Selected Environmental Factors

Canonical correspondence analysis (CCA) based on the species data and environmental variables showed that the first and second axes explained 27.6 % of the variance in the species data and 87.2 % of the variance in the species and environment relationship. The first axis explained 18.3 % of the variance in the species data and 57.7 % of the variance in the species and environmental relationship. The eigenvalues of axes 1, 2, 3 and 4 were 0.395, 0.202, 0.087 and 0.369, respectively.

**Fig. 3:**
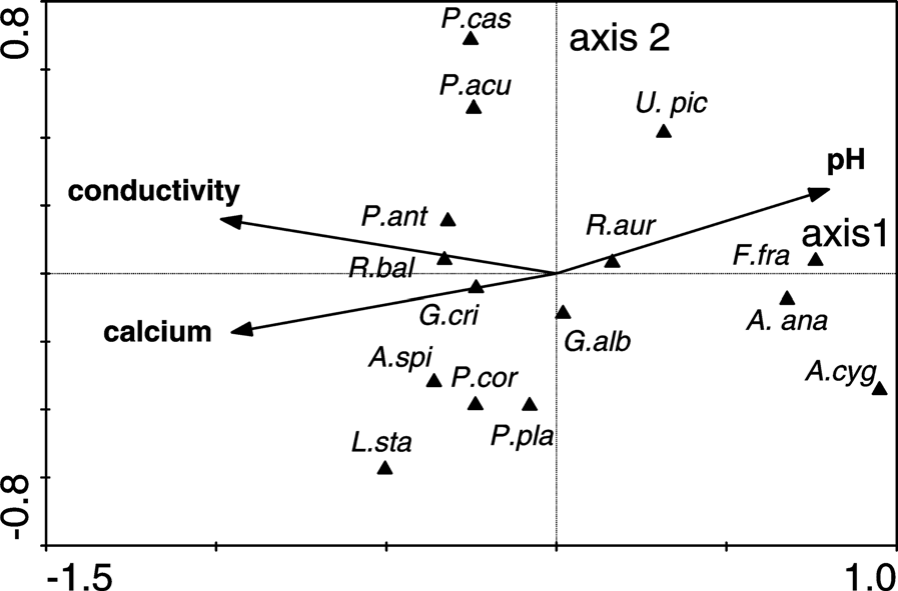
Ordination diagram (biplot) based on the canonical correspondence analysis (CCA) of the Mollusca data and environmental variables. *Long arrows*, which represent selected (statistically significant) environmental variables, emphasise their impact on the structure of the mollusc communities in the mining subsidence reservoirs

Conductivity, pH and the concentration of calcium were the parameters most associated (statistically significant according to the forward selection results) with the distribution of mollusc species (Fig. 3). CCA analysis showed that *Potamopyrgus antipodarum*, *Radix balthica*, *Physella acuta*, *Gyraulus crista* and *Pisidium casertanum* were associated with a higher conductivity and lower pH values. *Anodonta cygnea*, *Anodonta anatina* and *F. fragilis* were negatively influenced by these parameters of the water. *Anisus spirorbis*, *Planorbarius corneus*, *Planorbis planorbis* and *Lymnaea stagnalis* were positively influenced by the concentration of calcium in the water (Fig. 3).

The relationship between the species composition of Mollusca and the environmental variables was significant (Monte Carlo test of significance of the first canonical axis, *F*-ratio = 5.137, *p* value = 0.002; test of significance of the second canonical axis, *F*-ratio = 3.673, *p* value = 0.004; test of significance of all canonical axes, *F*-ratio = 3.547, *p* value = 0.002).

## Discussion

Many urban ponds in the contemporary landscape reflect an industrial legacy, but since they were created, a significant number have become important habitats for aquatic plants and animals, thus acquiring considerable conservation values (Rees 1997). Identifying new habitats in which freshwater molluscs occur is an important aspect of environmental and faunistic studies that provide a holistic view of environmental issues. This approach is particularly valuable in areas in which human activity has influenced the aquatic ecosystems, such as small artificial water bodies in urban industrial landscapes. According to Wood and Barker (2000), the pressures for development need to be balanced against the protection and enhancement of the environment, even if the environment is itself the result of past human activity. The mining subsidence reservoirs that were studied are small and shallow. In relation to size, water bodies support a disproportionately high richness of species and different community structures given their small size, and this pattern is well documented throughout Europe and globally (e.g. Froneman et al. 2001; Angélibert et al. 2004; Davies et al. 2008; Markwell and Fellows 2008).

The major threat to European freshwater molluscs is the loss and degradation of their habitats as a result of pollution and water quality decline, urbanisation, changes to the flow regime or the construction of roads as well as mine waste (Cuttelod et al. 2011). Freshwater ecosystems are gravely imperiled, particularly within most urbanised and industralised landscapes not only in Europe but also worldwide (Clements et al. 2006). However, our results showed the occurrence of 15 mollusc species, including unionid mussels and fingernail clams in the mining subsidence reservoirs that are located along the Trans-Regional Highway (T-R H). In comparison, the mining subsidence reservoirs of other locations support from 11 to 26 mollusc species (Lewin and Smoliński 2006; Michalik-Kucharz 2008; Kašovská et al. 2014; Strzelec et al. 2014), whereas 23 gastropod species were recorded in anthropogenic reservoirs that are isolated from urban areas (Spyra and Strzelec 2014). This survey found four bivalve species including *Anodonta cygnea*, a species that is protected by Polish legislation (Dz. U. 2014b). *Anodonta cygnea* is classified as Endangered (EN) according to the Polish Red Data Book of Animals and also as Near Threatened (NT) according to the European Red List of Non-marine Molluscs (Głowaciński and Nowacki 2004; Cuttelod et al. 2011). *Anodonta cygnea* primarily occurs in shallow eutrophic water bodies, oxbow lakes and lakes, slowly flowing lowland rivers, artificial ponds, canals and dam reservoirs (Abraszewska-Kowalczyk 2002; Lewin 2014). The major threats to *Anodonta cygnea* are habitat degradation and water pollution as well as the decline in the quality of the habitats in rivers and lakes. This survey uncovered the first occurrence of *Anodonta cygnea* in a mining subsidence reservoir in Upper Silesia as well as 11 out of 15 mollusc species that are included on the European Red List of Non-marine Molluscs as Least Concern (LC) (Cuttelod et al. 2011). Our survey revealed the occurrence of 11 gastropod species and 4 bivalve species in the mining subsidence reservoir located along T-R H in industrial areas as well as 6–11 mollusc species in particular reservoirs. In comparison, 4–14 gastropod species and 3 bivalve species were recorded in natural ponds in European countries and Canada and 1–10 in particular reservoirs (Pip 1986; Costil and Clement 1996; Baur and Ringeis 2002; Zealand and Jeffries 2009). Thus, the number of mollusc species recorded in these mining subsidence reservoirs is comparable to those recorded in natural ponds. According to Baur and Ringeis (2002), natural ponds support more mollusc species including the Red Lists species than running waters. Our results showed that *R. auricularia* was widespread in the mining subsidence reservoirs located along T-R H, whereas it was a rare species in natural ponds (Costil and Clement 1996). *R. balthica* and *G. albus*, which are able to establish themselves in a wide range of pond types, occurred both in the mining subsidence reservoirs located along T-R H and the natural ponds that were surveyed by Zealand and Jeffries (2009). In summary, the mining subsidence reservoir located along T-R H in industrial areas may create unique valuable ecosystems that contribute to the biodiversity of such areas.

The present survey found relatively high values of conductivity, hardness, concentration of chlorides and sulphates, especially in the two mining subsidence reservoirs that are located closest to the Trans-Regional Highway (T-R H). Such high values of the physical and chemical parameters of the water are typical of the mining subsidence reservoirs of Upper Silesia (ways of supply, including the mine dewatering system) (Lewin 2012; Strzelec et al. 2014). The construction of highways and transportation routes has an influence on the impairment of the quality of the water including an increase in conductivity and alkalinity as well as the concentrations of chlorides, calcium, ammonium and iron (Benbow and Merritt 2004; Hedrick et al. 2010). Our survey showed that the ranges of some water quality parameters were much higher than the ranges recommended for aquatic life. For example, the recommended values of chlorides and sulphates for freshwater organisms are below 11 mg Cl^−^ dm^−3^ and 100 mg SO_4_^2−^ dm^−3^ (Hedrick et al. 2010).

Our results of the CCA ordinations revealed that among the environmental factors, conductivity, pH and the concentration of calcium in the water were positively correlated with the structure of mollusc communities in the mining subsidence reservoirs. This finding is consistent with the previous surveys of Lewin (2012), Spyra and Strzelec (2014) and Kašovská et al. (2014), who consider these factors to be the most predictive that influence the distribution of mollusc species. Moreover, Clements et al. (2006) considered pH and the concentration of calcium in the water to be the most significant parameters related to the distribution of molluscs in urban area. This research found that alien species, *Potamopyrgus antipodarum* and *Physella acuta*, were associated with higher conductivity values, whereas *F. fragilis* as well as native species e.g. *Anodonta anatina* and *G. albus* were negatively influenced by this parameter of the water. The New Zealand mud snail, *Potamopyrgus antipodarum*, a successful invader of freshwater and brackish habitats in Australia, Europe, Japan, North America and western Asia, occurs more frequently in aquatic sites that have multiple land uses in their catchment area as compared to sites with low-impact human activities (Schreiber et al. 2003). Both *Physella acuta* and *Potamopyrgus antipodarum* tolerate a relatively high conductivity of the water (up to 6400 and 7390 μS cm^−1^, respectively) (Schreiber et al. 2003; Kefford et al. 2004; Zalizniak et al. 2009). *Potamopyrgus antipodarum* occurred in the mining subsidence reservoirs with the highest conductivity (Lewin 2012; Kašovská et al. 2014). The present results are consistent with the survey of Kašovská et al. (2014), who showed an association between *Physella acuta* and *Potamopyrgus antipodarum* and water conductivity above 2000 μS cm^−1^ whereas *Anodonta anatina* and *G. albus* were associated with lower conductivity values. According to Verbrugge et al. (2012), the maximum salinity tolerance ranged from 0.5 to 19.0‰ for the native mollusc species, while it ranged 1.0–28.0‰ for alien mollusc species. They showed that both *Potamopyrgus antipodarum* and *Physella acuta* occur in water environments with a maximum salinity of 33.5‰ (the field) whereas *F. fragilis* occurs in water environments with a maximum salinity of 1‰. In comparison, our survey showed the occurrence of *Potamopyrgus antipodarum*, *Physella acuta* and *F. fragilis* in reservoirs with conductivity of 1920 μS cm^−1^, which corresponds to a salinity of about 1‰.

This research also found (CCA analysis) that *L. stagnalis*, *Planorbarius corneus* and *Planorbis planorbis* were the species correlated with a higher concentration of calcium in the water. These results support the survey of Briers (2003), which states that these species are calciphiles and therefore require an environmental calcium concentration of 20 mg Ca dm^−3^ or greater. In contrast to noncalciphiles, they are only able to exploit a narrow range of calcium concentrations. They utilise habitats that have a restricted distribution, thus resulting in smaller ranges. *L. stagnalis*, *Planorbarius corneus* and *Planorbis planorbis* were eudominants or dominants in the mining subsidence reservoir that had a higher concentration of calcium (reservoir 3).

According to Williams et al. (2004), the rich biodiversity found in water bodies arises because individual ponds typically support surprisingly different communities to one another, even in water bodies that are close to each other such as the mining subsidence reservoirs that were studied in this research. The differences in the distribution of molluscs among the reservoirs can be explained by both the local conditions (e.g. the physical and chemical parameters of the water) within the reservoirs and the spatial patterns (e.g. distance between ponds) (Zealand and Jeffries 2009). However, separating the local from the spatial patterns is difficult because the two are often linked and acting together. These results showed that the differences in the distribution of molluscs among the mining subsidence reservoirs can be explained by the conductivity gradient, pH and the concentration of calcium in the water. According to Pip (1986), the occurrence of particular mollusc species in particular ponds depends on many environmental factors including environmental suitability and stability (the physical and chemical parameters of the water, the densities of other mollusc species that are already present in reservoirs, the occurrence of macrophytes, the availability of food and the presence of predators and parasites). The occurrence of mussel species in particular reservoirs can be the result of stocking with fish that are appropriate for glochidium development. Unionoidea require host fishes in order to complete their successful recruitment. Our survey showed the occurrence of mussel species in two of the four mining subsidence reservoirs. These results can be explained by the presence of obligatory host fish species that are required by Unionoidea in only two mining subsidence reservoirs. The differences in the distribution of mollusc species among isolated reservoirs may also be explained by the element of chance because it can influence molluscs dispersal, migration, population fluctuations, etc. (Pip 1986).

The loss of many pond habitats and its potential impact on biodiversity have been well documented on both continental and regional scales (Heath and Whitehead 1992; Blaustein and Wake 1995) although measures to protect water bodies have yet to be established in most locations. This is especially important in industrial areas where many of the natural water bodies have disappeared due to the development of industry. The aesthetic, recreation potential and conservation values of freshwater reservoirs in urban, industrial and rural landscapes have been widely acknowledged (Boothby et al. 1995; House 1996; House and Fordham 1997; Rees 1997). The formation of reservoirs along transportation routes is a phenomenon that can be increasingly observed. This is particularly important for the biodiversity of freshwater fauna. The results of this survey showed that the mining subsidence reservoirs that are located in urbanised and industrialised areas provide refuges for rare and legally protected species and that they play an essential role in the dispersal of alien species as well. According to Wood and Barker (2000), industrial water bodies constitute biological oases for many species of flora and fauna in largely artificial and sometimes contaminated habitats. The results of the study of Le Viol et al. (2009) and Colino-Rabanal and Lizana (2012) also suggest that highway ponds may contribute to the biodiversity of the pond network at a regional scale in altered landscapes. These types of industrial reservoirs have been suggested as potentially constituting corridors and refuges as they may provide alternative stable habitats in which species can complete their life cycles when their habitats have been degraded. It is necessary to undertake further surveys of ponds in urban and industrial settings in order to develop comprehensive baseline data regarding water quality and floral and faunal biodiversity to aid in the management and conservation of the entire pond resource. This also applies to the water reservoirs that are increasingly being formed along the transportation routes and highways.

## Conclusions

The results of this survey showed that the mining subsidence reservoirs that are located in urbanised and industrialised areas provide refuges for rare and legally protected species and that they play an essential role in the dispersal of alien species as well. Mining subsidence reservoirs may create a unique valuable ecosystem that contributes to the natural diversity of industrial areas. Therefore, conservation planning, which traditionally focuses on the protection of natural habitats, should also take anthropogenic reservoirs including the mining subsidence reservoirs located along transportation routes and highways into consideration.

## Abbreviations

P.ant: *Potamopyrgus antipodarum*
R.aur: *Radix auricularia*
R.bal: *Radix balthica*
L.sta: *Lymnaea stagnalis*
P.acu: *Physella acuta*
P.cor: *Planorbarius corneus*
F.fra: *Ferrissia fragilis*
P.pla: *Planorbis planorbis*
A.spi: *Anisus spirorbis*
G.alb: *Gyraulus albus*
G.cri: *Gyraulus crista*
U.pic: *Unio pictorum*
A.cyg: *Anodonta cygnea*
A.ana: *Anodonta anatina*
P.cas: *Pisidium casertanum*
